# Supplementary material for: Model-guided engineering of DNA sequences with predictable site-specific recombination rates
Source: Nat Commun. 2022 Jul 20;13:4152. doi: 10.1038/s41467-022-31538-3 (PMC9300676; doi:10.1038/s41467-022-31538-3)
Supplement: Supplementary file 1 — Supplementary Information [file 41467_2022_31538_MOESM1_ESM.pdf]

Supplementary Information for:

Model-guided engineering of DNA sequences with predictable site-specific recombination rates

Qiuge Zhang[1], Samira M. Azarin[1], Casim A. Sarkar[2,*]

[1] Department of Chemical Engineering and Materials Science, University of Minnesota, Minneapolis, Minnesota 55455, United States

[2] Department of Biomedical Engineering, University of Minnesota, Minneapolis, Minnesota 55455, United States

*Address correspondence to Casim A. Sarkar (csarkar@umn.edu)

Supplementary Tables

Supplementary Table 1. Oligonucleotides used in this study

Purpose	Name	Sequence (5' → 3')
Linear DNA library	F-library upstream	AGGCCTTGGAGCCGTACATG
	F-library downstream	CCCGGGAAGCTTTTTAAGAAGGAGATATACATATGAGCA<br>AAGGAGAAGAACTTTT
	R-library downstream	TCCGTTTGTAGCATCACC
	R-library1 upstream	CTTAAAAAGCTTTCGT**GGTTTGNNNNNNNNNNCACCGCG**<br>**G**TCTCAGTGGT
	R-library2 upstream	CTTAAAAAGCTTTCGT**GGTTTNTNNNNTNNACNACNGNG**<br>**G**TCTCAGTGGTGTACGG
	R-WT upstream	CTTAAAAAGCTTTCGT**GGTTTGTCTGGTCAACCACCGCG**<br>**G**TCTCAGTGGT
Bxb1 purification plasmid: pET22-bxb1	F-bxb1	GATATACATATGGTGAGAGCCCTGGTAG
	R-bxb1	GTGGTGGGTCTCCTCGAGCGACATCCCGGTGTGTAG
MiniSeq amplicon preparation	F-first step	TCGTCGGCAGCGTCAGATGTGTATAAGAGACAGTGATCC<br>TGACGACGGAGA
	R-first step	GTCTCGTGGGCTCGGAGATGTGTATAAGAGACAGAAAAA<br>GCTTTCGTGGTTT
qPCR quantification	F-qPCR	TTGTGCCCATTAACATCACC
	R-qPCR	ATGATCCTGACGACGGAGA
Variants for *in vitro* validation	R-C14A	CTTAAAAAGCTTTCGT**GGTTTGTCTGTTCAACCACCGCG**<br>**G**TCTCAGTGGT
	R-G5C	CTTAAAAAGCTTTCGT**GGTTTGTCTGGTCAACCACGGCG**<br>**G**TCTCAGTGGT
	R-A16C	CTTAAAAAGCTTTCGT**GGTTTGTCGGGTCAACCACCGCG**<br>**G**TCTCAGTGGT
	R-G12C	CTTAAAAAGCTTTCGT**GGTTTGTCTGGTGAACCACCGCG**<br>**G**TCTCAGTGGT
	R-G5A	CTTAAAAAGCTTTCGT**GGTTTGTCTGGTCAACCACTGCG**<br>**G**TCTCAGTGGT
	R-G17A	CTTAAAAAGCTTTCGT**GGTTTGTTTGGTCAACCACCGCG**<br>**G**TCTCAGTGGT
	R-3T, 5A, 12C, 14T, 17A	CTTAAAAAGCTTTCGT**GGTTTGTTTGATGAACCACTGTG**<br>**G**TCTCAGTGGT
	R-5T, 12A, 17A	CTTAAAAAGCTTTCGT**GGTTTGTTTGGTTAACCACAGCG**<br>**G**TCTCAGTGGT
	R-5A, 8T, 12C, 14T, 17A	CTTAAAAAGCTTTCGT**GGTTTGTTTGATGAACAACTGCG**<br>**G**TCTCAGTGGT
pUC-gfp/mcherry for *in vivo* validation	F-mCherry	CCCGGGTCTAGAGCGAGTCAG
	R-mCherry	GGGCCCGGATCCTTTAAGAAGGAGAT
	F-attB	GGGCCCGGATCCTCGGCCGGCTTGTC

	R-attP	CCCGGGAAGCTTTCGTGGTTTGTCTGGTCAACCACCGCGGTCTCAGTGGTGTACGGTACAAACCCGCTAGCAGCTGTTTCCTGTGTGAAATTGTTATCCG
	F-GFP	CCCGGGAAGCTTTTTAAGAAGGAGATATACATATGAGCAAAGGAGAAGAACTTTT
	R-GFP	GGGCCCGGTACCTAAAACGACGGCCAGTGAATTCGAGCTCCAAAAAACCCCTCAAGACCC
	F-pUC vector	GGGCCCGGTACCCAACGTCGTGACTGGG
	R-pUC vector	CCCGGGTCTAGATGCGCTCGGTCGTTC
Bxb1 donor plasmid for *in vivo* validation: p15A-P_{BAD}-bxb1	F-PBAD	ACCTGGTGCACGGCAGATACACTTGCTG
	R-PBAD	CTTGAGGAATTCTTGAAGACGAAAGGGC
	F-bxb1 (PBAD)	TCGTCTTCAAGAATTCCTCAAGAAGATCCTTTGATC
	R-bxb1 (PBAD)	TCTGCCGTGCACCAGGTGTCTAGAAGAGACAC
KanR/CmR reporter plasmid for preliminary selection *in vivo*: p15A-P_{cons}-kanR/cmR	F-KanR	GGTGTTGGTACCTCGGCCGGCTTGTCGACGACGGCGGTCTCCGTCGTCAGGATCATCCGGGCAGGAGATATACATATGATGAGCCATATTCAACGG
	R-KanR	GCGTAAAAGCTTTTAGAAAAACTCATCGAGC
	F-CmR	TTCTAAAAGCTTTTACGCCCCGCCCTGCCAC
	R-CmR	CAATTGGGATCCAGGAGATATACATATGATGGAGAAAAAAATCACTGG
Bxb1 donor plasmid for preliminary selection *in vivo*: pUC-bxb1	F-bxb1 (pUC)	TCCCCGGGTACCGTGAGAGCCCTGGTAG
	R-bxb1 (pUC)	TGCACCATATGCTTACGACATCCCGGTGTGTAG

Supplementary Table 2. Plasmids used in this study

Plasmid	Name	Origin of replication	Promoter	Antibiotic resistance
Bxb1 donor for *in vivo* validation	p15A-P_{BAD}-bxb1	p15A (low copy)	Arabinose promoter	Chloramphenicol
Bxb1 donor for *in vivo* preliminary selection	pUC19-P_{BAD}-bxb1	pUC19 (high copy)	Arabinose promoter	Carbenicillin
Bxb1 purification	pET22-P_{T7}-bxb1	pET22 (medium copy)	T7 promoter	Carbenicillin
GFP/mCherry reporter for *in vivo* validation	pUC19-attB-P_{Lac}-attP-gfp/mcherry	pUC19 (high copy)	Lactose promoter	Carbenicillin
KanR/CmR reporter for *in vivo* preliminary selection	p15A-attB-P_{cons}-attP-kanR/cmR	p15A (low copy)	Constitutive promoter	Kanamycin or Chloramphenicol

Supplementary Table 3. Strains used in this study

Purpose	Host cell	Plasmid 1	Plasmid 2
In vivo validation of attP variants	TOP10 pro	p15A-P_{BAD}-bxb1	pUC19-attB-P_{Lac}-attP-gfp/mcherry
Negative control for *in vivo* validation of attP variants	TOP10 pro	--	pUC19-attB-P_{Lac}-attP-gfp/mcherry
Preliminary library 1 selection *in vivo*	TOP10 pro	pUC19-P_{BAD}-bxb1	p15A-attB-P_{cons}-attP-kanR/cmR
Bxb1 protein purification	BL21(DE3)	pET22-P_{T7}-bxb1	--

Supplementary Table 4. Sequences of library 1 and library 2

	Position																							
	1	2	3	4	5	6	7	8	9	10	11	12	13	14	15	16	17	18	19	20	21	22	23	24
WT	C	C	G	C	G	G	T	G	G	T	T	G	A	C	C	A	G	A	C	A	A	A	C	C
Library 1	C	C	G	C	G	G	T	G	N	N	N	N	N	N	N	N	N	N	C	A	A	A	C	C
Library 2	C	C	N	C	N	G	T	N	G	T	N	N	A	N	N	N	N	A	N	A	A	A	C	C

Supplementary Table 5. Nucleotide enrichment of library 1 after selection with different Bxb1 concentrations

Selection condition	Position	9	10	11	12	13	14	15	16	17	18
	WT	G	T	T	G	A	C	C	A	G	A
Naïve library	A	0.25	0.25	0.23	0.25	0.25	0.23	0.24	0.22	0.23	0.28
	C	0.22	0.27	0.28	0.26	0.29	0.27	0.26	0.27	0.28	0.30
	G	0.28	0.24	0.23	0.25	0.21	0.25	0.26	0.26	0.26	0.24
	T	0.25	0.25	0.26	0.24	0.25	0.25	0.24	0.23	0.23	0.18
300 nM, 1h	A	0.27	0.14	0.18	0.24	**0.28**	0.15	0.16	**0.25**	0.27	**0.35**
	C	0.17	0.35	0.37	0.39	0.31	**0.47**	**0.46**	0.37	0.35	0.31
	G	**0.42**	0.07	0.21	**0.21**	0.22	0.15	0.22	0.22	**0.20**	0.24
	T	0.14	**0.45**	**0.24**	0.16	0.20	0.22	0.17	0.17	0.18	0.10
300 nM, 5min	A	0.24	0.09	0.17	0.23	**0.29**	0.11	0.13	**0.26**	0.28	**0.38**
	C	0.12	0.26	0.34	0.40	0.29	**0.53**	**0.49**	0.36	0.33	0.29
	G	**0.52**	0.05	0.23	**0.21**	0.22	0.13	0.24	0.23	**0.21**	0.24
	T	0.12	**0.60**	**0.26**	0.15	0.20	0.23	0.14	0.15	0.19	0.09
100 nM, 5min	A	0.23	0.08	0.17	0.24	**0.29**	0.11	0.12	**0.27**	0.30	**0.44**
	C	0.11	0.24	0.34	0.39	0.29	**0.51**	**0.49**	0.36	0.31	0.25
	G	**0.55**	0.04	0.23	**0.22**	0.21	0.13	0.24	0.22	**0.22**	0.24
	T	0.11	**0.64**	**0.26**	0.15	0.21	0.24	0.15	0.15	0.17	0.07
50 nM, 5min	A	0.22	0.07	0.16	0.25	**0.30**	0.11	0.12	**0.28**	0.30	**0.47**
	C	0.09	0.22	0.33	0.38	0.28	**0.51**	**0.49**	0.35	0.31	0.24
	G	**0.59**	0.04	0.24	**0.23**	0.21	0.14	0.24	0.22	**0.22**	0.23
	T	0.10	**0.67**	**0.27**	0.14	0.20	0.24	0.15	0.15	0.16	0.06
25 nM, 5min	A	0.18	0.06	0.15	0.26	**0.31**	0.12	0.13	**0.32**	0.32	**0.52**
	C	0.07	0.17	0.32	0.36	0.27	**0.50**	**0.49**	0.34	0.28	0.21
	G	**0.67**	0.03	0.24	**0.24**	0.22	0.14	0.23	0.20	**0.24**	0.21
	T	0.09	**0.74**	**0.29**	0.14	0.19	0.25	0.16	0.14	0.15	0.05
10 nM, 5min	A	0.19	0.06	0.15	0.26	**0.32**	0.13	0.13	**0.33**	0.32	**0.52**
	C	0.08	0.18	0.32	0.35	0.26	**0.49**	**0.49**	0.33	0.28	0.22
	G	**0.65**	0.03	0.24	**0.25**	0.21	0.14	0.22	0.20	**0.23**	0.21
	T	0.09	**0.72**	**0.30**	0.14	0.20	0.24	0.16	0.14	0.17	0.06
5 nM, 5min	A	0.18	0.08	0.15	0.23	**0.34**	0.12	0.13	**0.32**	0.28	**0.52**
	C	0.10	0.20	0.30	0.35	0.26	**0.49**	**0.52**	0.32	0.22	0.22
	G	**0.62**	0.04	0.20	**0.25**	0.21	0.13	0.20	0.21	**0.24**	0.20
	T	0.10	**0.67**	**0.35**	0.14	0.19	0.26	0.16	0.15	0.16	0.06
4 nM, 5min	A	0.17	0.06	0.14	0.24	**0.37**	0.12	0.13	**0.34**	0.33	**0.56**
	C	0.07	0.16	0.29	0.35	0.26	**0.48**	**0.52**	0.33	0.26	0.19
	G	**0.69**	0.03	0.22	**0.25**	0.19	0.13	0.19	0.21	**0.22**	0.19
	T	0.07	**0.75**	**0.35**	0.16	0.18	0.27	0.16	0.12	0.19	0.05
3 nM, 5min	A	0.19	0.07	0.14	0.27	**0.39**	0.15	0.15	**0.34**	0.35	**0.49**
	C	0.10	0.19	0.29	0.37	0.24	**0.50**	**0.47**	0.33	0.29	0.22
	G	**0.63**	0.04	0.21	**0.24**	0.20	0.14	0.21	0.19	**0.21**	0.22
	T	0.08	**0.70**	**0.36**	0.14	0.18	0.31	0.17	0.14	0.15	0.07

Supplementary Table 6. Nucleotide enrichment of library 2 after selection with different Bxb1 concentrations

Selection condition	Position	3	5	8	11	12	14	15	16	17	19
	WT	G	G	G	T	G	C	C	A	G	C
Naïve library	A	0.23	0.25	0.23	0.25	0.24	0.23	0.24	0.22	0.23	0.23
	C	0.28	0.27	0.28	0.26	0.24	0.27	0.26	0.27	0.28	0.30
	G	0.24	0.24	0.23	0.25	0.21	0.25	0.26	0.26	0.26	0.24
	T	0.25	0.25	0.26	0.24	0.24	0.25	0.24	0.23	0.23	0.22
300 nM, 1h	A	0.26	0.24	0.23	0.23	0.23	0.11	0.14	**0.22**	0.23	0.06
	C	0.26	0.23	0.28	0.29	0.39	**0.47**	**0.40**	0.30	0.29	**0.80**
	G	**0.26**	**0.25**	**0.17**	0.22	**0.21**	0.16	0.28	0.27	**0.28**	0.08
	T	0.23	0.29	0.33	**0.25**	0.17	0.26	0.19	0.21	0.21	0.06
300 nM, 5min	A	0.22	0.26	0.21	0.22	0.23	0.06	0.10	**0.23**	0.25	0.02
	C	0.21	0.22	0.28	0.28	0.41	**0.54**	**0.42**	0.29	0.26	**0.91**
	G	**0.30**	**0.24**	**0.16**	0.24	**0.21**	0.11	0.33	0.29	**0.28**	0.04
	T	0.28	0.28	0.35	**0.27**	0.15	0.28	0.16	0.20	0.22	0.02
100 nM, 5min	A	0.21	0.26	0.20	0.21	0.22	0.06	0.10	**0.23**	0.25	0.02
	C	0.20	0.22	0.27	0.28	0.41	**0.54**	**0.42**	0.29	0.26	**0.94**
	G	**0.31**	**0.24**	**0.16**	0.24	**0.21**	0.11	0.33	0.29	**0.28**	0.03
	T	0.28	0.28	0.37	**0.28**	0.15	0.29	0.16	0.19	0.21	0.02
50 nM, 5min	A	0.19	0.26	0.19	0.20	0.23	0.05	0.09	**0.22**	0.25	0.01
	C	0.19	0.22	0.28	0.28	0.40	**0.55**	**0.42**	0.29	0.28	**0.96**
	G	**0.33**	**0.24**	**0.16**	0.24	**0.22**	0.11	0.33	0.30	**0.27**	0.02
	T	0.28	0.28	0.37	**0.28**	0.15	0.28	0.16	0.19	0.20	0.01
25 nM, 5min	A	0.16	0.27	0.18	0.18	0.22	0.06	0.10	**0.25**	0.28	0.01
	C	0.17	0.20	0.25	0.28	0.40	**0.53**	**0.42**	0.29	0.25	**0.97**
	G	**0.38**	**0.21**	**0.15**	0.25	**0.22**	0.12	0.33	0.29	**0.28**	0.01
	T	0.30	0.31	0.42	**0.29**	0.15	0.29	0.16	0.18	0.20	0.01
10 nM, 5min	A	0.16	0.28	0.17	0.18	0.22	0.06	0.10	**0.25**	0.28	0.01
	C	0.17	0.19	0.24	0.27	0.41	**0.53**	**0.42**	0.29	0.25	**0.96**
	G	**0.38**	**0.21**	**0.15**	0.25	**0.23**	0.12	0.33	0.28	**0.28**	0.01
	T	0.30	0.32	0.44	**0.30**	0.15	0.29	0.15	0.17	0.19	0.01
5 nM, 5min	A	0.17	0.26	0.18	0.18	0.22	0.06	0.09	**0.24**	0.27	0.01
	C	0.17	0.22	0.25	0.27	0.40	**0.53**	**0.42**	0.29	0.27	**0.95**
	G	**0.36**	**0.21**	**0.15**	0.24	**0.23**	0.12	0.32	0.29	**0.27**	0.02
	T	0.30	0.31	0.42	**0.30**	0.15	0.28	0.16	0.18	0.19	0.02
4 nM, 5min	A	0.13	0.27	0.15	0.15	0.20	0.07	0.10	**0.31**	0.35	0.01
	C	0.16	0.18	0.19	0.26	0.41	**0.51**	**0.43**	0.27	0.22	**0.96**
	G	**0.42**	**0.17**	**0.13**	0.25	**0.25**	0.13	0.32	0.27	**0.26**	0.01
	T	0.28	0.38	0.53	**0.34**	0.14	0.28	0.16	0.15	0.17	0.02
3 nM, 5min	A	0.13	0.27	0.15	0.14	0.20	0.07	0.10	**0.31**	0.35	0.01
	C	0.17	0.17	0.19	0.26	0.41	**0.52**	**0.42**	0.27	0.22	**0.96**
	G	**0.43**	**0.17**	**0.13**	0.25	**0.25**	0.13	0.32	0.27	**0.26**	0.01
	T	0.28	0.39	0.53	**0.35**	0.14	0.28	0.16	0.15	0.17	0.01
2 nM, 5min	A	0.08	0.32	0.11	0.14	0.20	0.06	0.11	**0.29**	0.32	0.01
	C	0.09	0.14	0.20	0.25	0.39	**0.54**	**0.42**	0.31	0.24	**0.97**
	G	**0.57**	**0.12**	**0.13**	0.29	**0.27**	0.12	0.33	0.27	**0.27**	0.01
	T	0.25	0.42	0.56	**0.32**	0.14	0.27	0.14	0.13	0.16	0.01
1 nM, 5min	A	0.10	0.24	0.09	0.15	0.18	0.09	0.14	**0.32**	0.29	0.01
	C	0.08	0.28	0.22	0.24	0.40	**0.53**	**0.41**	0.29	0.30	**0.97**
	G	**0.57**	**0.13**	**0.15**	0.28	**0.27**	0.14	0.30	0.21	**0.23**	0.01
	T	0.25	0.35	0.54	**0.33**	0.14	0.24	0.14	0.17	0.19	0.01

Supplementary Table 7. Conversion of DNA sequences into binary matrices

**Position**	**3**	**5**	**8**	**11**	**12**	**14**	**15**	**16**	**17**
**1 (WT reference)**	G	G	G	T	G	C	C	A	G
**2**	A	A	A	A	A	A	A	C	A
**3**	C	C	C	C	C	G	G	G	C
**4**	T	T	T	G	T	T	T	T	T

Example matrix for sequence GAGCGACGT:

**Sequence**	**G**	**A**	**G**	**C**	**G**	**A**	**C**	**G**	**T**
X_{ij}	**i=1**	**i=2**	**i=3**	**i=4**	**i=5**	**i=6**	**i=7**	**i=8**	**i=9**
**j=1**	1	0	1	0	1	0	1	0	0
**j=2**	0	1	0	0	0	1	0	0	0
**j=3**	0	0	0	1	0	0	0	1	0
**j=4**	0	0	0	0	0	0	0	0	1

Supplementary Figures

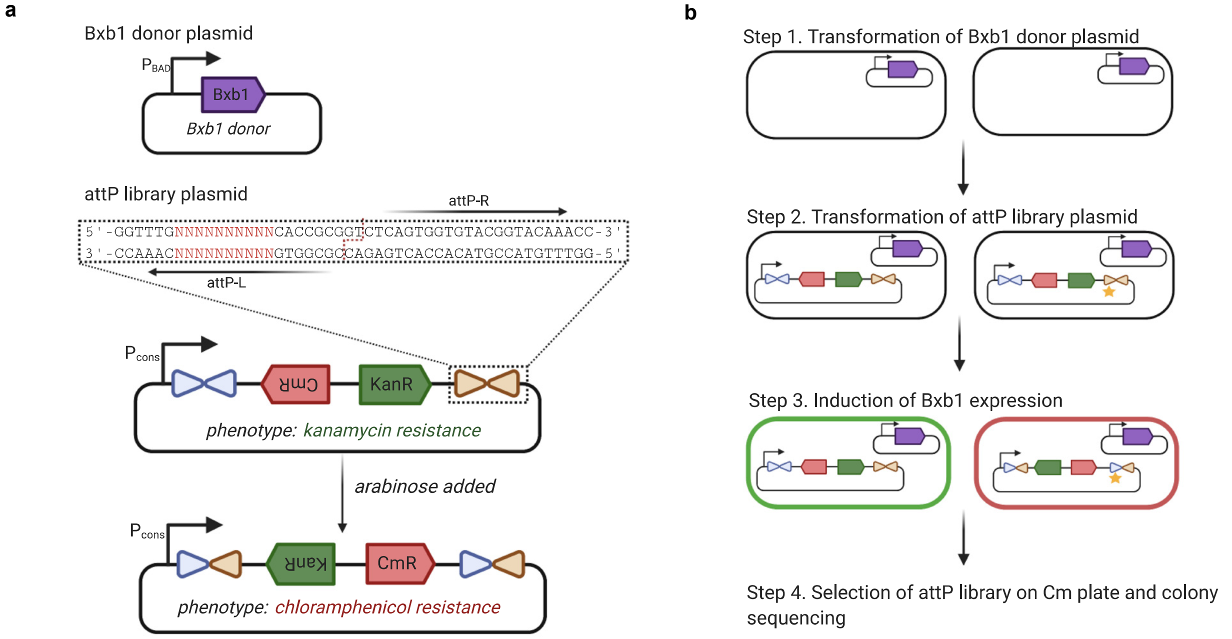

c

Colony number	Sanger sequencing result
1	GTCCCCAAAA
2	GTGTATCGGC
3	GTTCACGAAA
4	GTTAGTAAGA
5	GTAATACTAA
6	GTTTCCCCGA
7	GTCGATGACG
8	GTGCAACCAG
9	GTTATGCTGA
10	GTAACCCATA
11	GTTACCACTA
12	GTGTGCGACC
13	ATTAATCAGA
14	GTCCGTTGTA
15	GTCGCACACA
16	GTGGTCCGCA
17	GCTCAGTCCA
18	GTGCCGCGAC
19	GTGCTCTGCG
20	GTTGACTCAA

Supplementary Figure 1. *In vivo* selection in *E. coli* with library 1. **a** Diagrams of the Bxb1 donor plasmid (top) and KanR/CmR reporter plasmid (middle). The attP-L half-site library 1 was inserted into the KanR/CmR reporter plasmid for selection. The kanamycin resistance gene and the chloramphenicol resistance gene were placed in opposite directions and downstream of a constitutive promoter. Before addition of L-arabinose to induce Bxb1 recombinase expression, the reporter plasmid confers a kanamycin-resistant phenotype (green). After addition of L-arabinose, a reporter plasmid possessing a functional and efficient variant of the attP-L half-site switches phenotype from kanamycin-resistant (green, middle) to chloramphenicol resistance (red, bottom), and can therefore be selected on LB-agar plates with chloramphenicol. **b** Dual-step transformation of the Bxb1 donor plasmid and the KanR/CmR reporter plasmid, and the phenotype conversion from kanamycin resistance (green) to chloramphenicol resistance (red) after inducible expression of the Bxb1 recombinase. **c** Sanger sequencing results for single colonies selected on chloramphenicol-containing LB-agar plates. Supplementary Figs. 1a and 1b were created with BioRender.com.

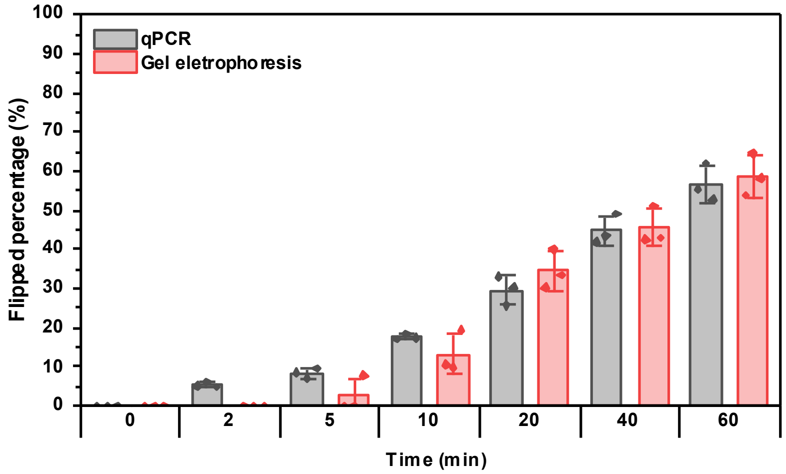

Supplementary Figure 2. Comparison of quantitative methods for measuring DNA inversion. The DNA recombination assay was performed by mixing 20 nM Bxb1 and 10 nM DNA in a 50 µl reaction. The reaction was incubated at 30 °C and terminated by heating after different times. For qPCR quantification, the reaction mixture was diluted and added to a qPCR reaction as a template, then the flipped percentage was calculated based on the C_q value. For gel electrophoresis, HindIII-HF was added to the cooled re action buffer to digest the DNA. After incubation at 37 °C for 1 h, 15 µl of the digest was subjected to gel electrophoresis. The percentage of flipped DNA was then calculated based on gel quantification of the band intensities of flipped and unflipped DNA. When the DNA flipping reaction duration was 2 min or less, flipped DNA was not detectable by gel quantification. Data are the mean values of three technical replicates, with error bars representing standard deviations. Source data are provided as a Source Data file.

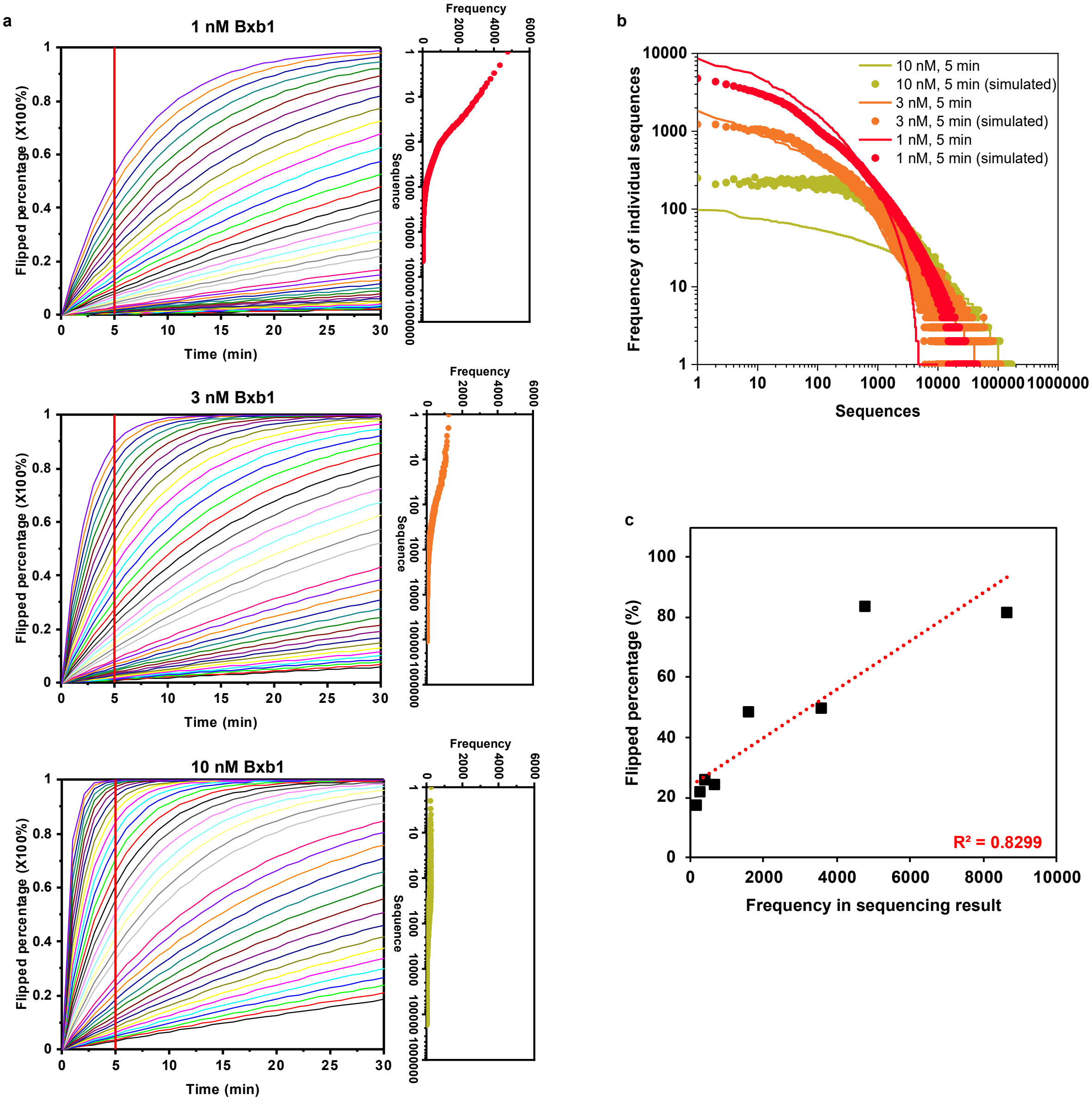

Supplementary Figure 3. Correlation between the frequency in NGS results and the DNA flipping rate constant k_{flip} for a given sequence. **a** (*left*) Simulations of flipped percentage for each possible sequence in library 2 were performed at a total DNA concentration of 10 nM and different Bxb1 concentrations (1 nM, 3 nM, and 10 nM) with first-order reaction kinetics (see Supplementary Method 2), with a representative subset of the response curves shown here. (*right*) At 5 min (corresponding to the vertical red lines in the plots on the left), the frequencies of flipped sequences were computed and plotted as a function of sequence number. **b** Comparison of frequency profiles of the experimental results from NGS with the simulated results based on predicted flipping rate constants k_{flip}. **c** Correlation between the flipped percentage in inversion assays conducted on eight individual sequences and the frequency of each of these sequences in the NGS results. For the given sequencing depth (1,000,000 reads per library), NGS is capable of identifying attP variants that yield at least a ~20% flipped percentage in these assay conditions. Source data are provided as a Source Data file.

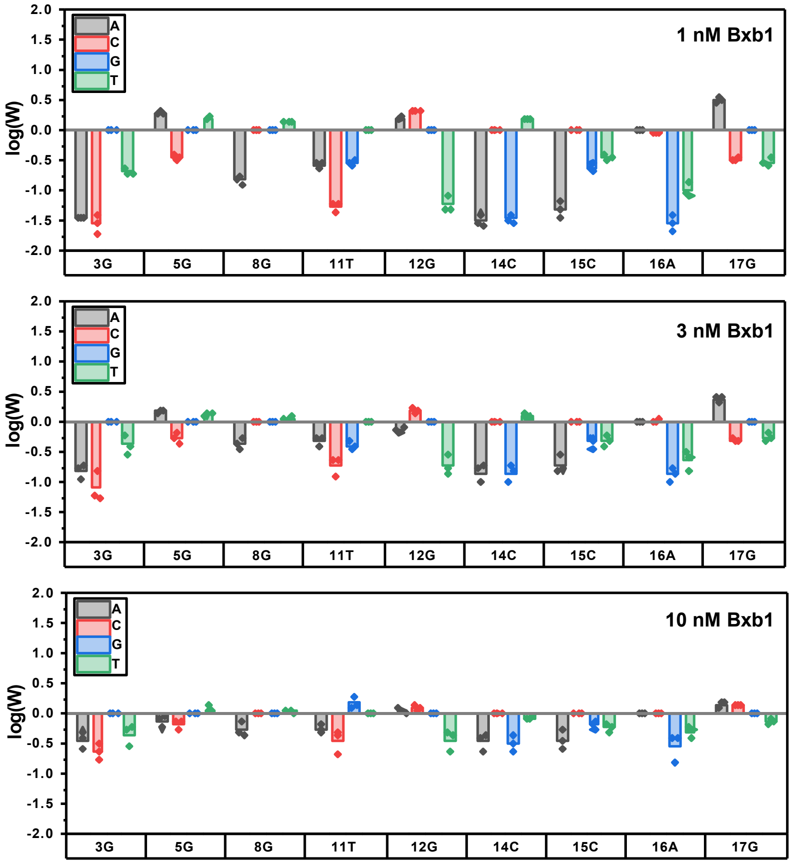

Supplementary Figure 4. Comparison of fitted weight scores for different Bxb1 concentrations. Weight score values, W, were generated from three sets of 200 training sequences, randomly chosen from the 3000 most enriched sequences. Three 200-sequence training data sets were randomly chosen for each Bxb1 concentration. Data are the mean values from three randomly selected sequence sets. Source data are provided as a Source Data file.

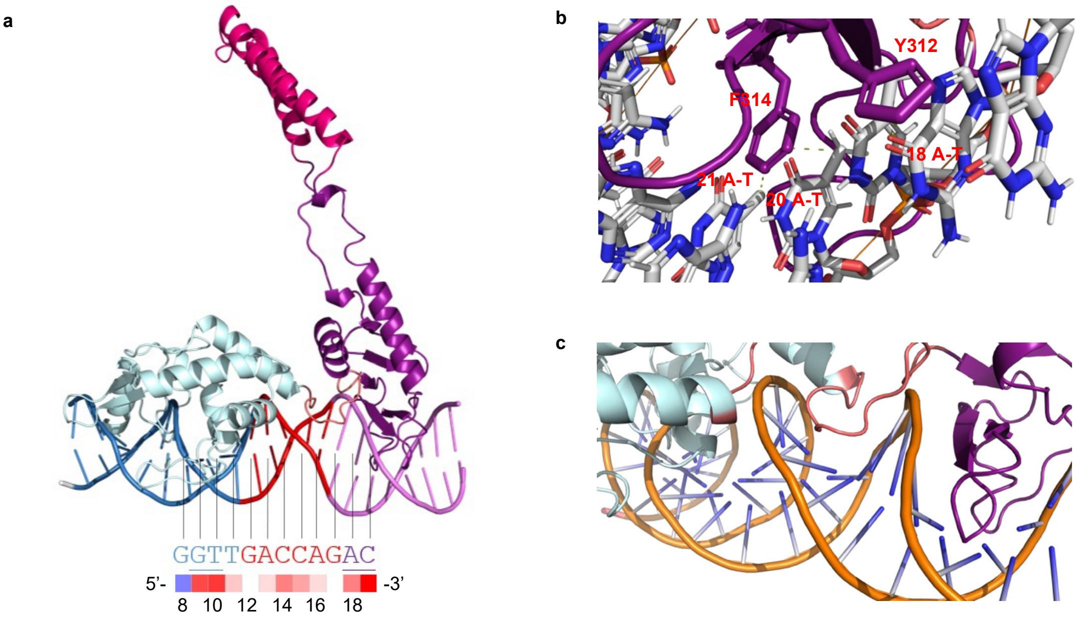

Supplementary Figure 5. Posited interactions between Bxb1 and attP-L based on fitted weight scores and homology modeling. **a** The Bxb1-DNA complex structure was simulated using SWISS-MODEL (https://swissmodel.expasy.org/)[1] and NPDock (http://genesilico.pl/NPDock)[2]. **b** Interactions between side chains in the Bxb1 zinc ribbon domain and bound, conserved nucleotides. **c** Interactions between side chains in the Bxb1 linker domain and bound, substitutable nucleotides.

We sought to gain biological insights from the sequence weight scores by comparing our identified conserved positions, positive substitutions, and negative substitutions with previous studies on large serine recombinases.[3] Although a crystal structure of the Bxb1-DNA complex was not available, there was a co-crystal structure of DNA bound by the integrase A118, which is from the same LSR subgroup as Bxb1.[3] Since the C-terminal domain is moderately conserved in LSRs, we used the structure of the A118-attP-L half-site complex (PDB: 4KIS) as a template to simulate Bxb1-DNA interactions with SWISS-MODEL and NPDock. As shown in Supplementary Fig. 5a, the recombinase domain (RD) and the zinc ribbon domain (ZD) form extensive direct contacts with the 24 bp of the attP-L half-site.

Nucleotide positions 18-24 (purple) at the site terminus are highly conserved among the four half-sites. We therefore hypothesized that these sites are likely essential for function, which was supported by our initial library selections. In addition, a previous study demonstrated that the outside flanking sequences were important for Bxb1 enzyme binding.[4] According to the published report on the A118-attP crystal structure complex,[3] hydrophobic interactions between the side chains of T288(β11), Y295(β12) and Y297(β12) and the 5-methyl groups of 23 A-T, 24 A-T, and 25 A-T are functionally important for protein binding to DNA. It was demonstrated that residues such as tyrosine can interact hydrophobically with the 5-methyl group on thymine, and the carbonyl group of aspartate residues can form hydrogen bonds with the amino group of the nucleic acid. Analogously, in the homology model of Bxb1-attP (Fig. S5b), 18 A-T, 20 A-T, and 21 A-T are suggested to form hydrophobic interactions with Y312 and F314 in the corresponding β-sheets and thus are likely highly conserved (which we experimentally demonstrated for 18A).

This structural model also suggests that nucleotides bound to RD (2-11, blue) and ZD-RD linker (12-16, red) are more tolerant to sequence substitutions. At positions 5, 12, and 17, some substitutions can even enhance the flipping rate. This improvement in activity could arise from higher protein-DNA binding affinity or any following steps in Fig. 1a. The highest weight score, for the G17A substitution, is also supported by a previous study on the Bxb1 attP site in which it was found that the binding affinity of the Bxb1 CTD for the attP-L half-site mutation G17A was twice that of the WT attP-L half-site (70 nM vs. 140 nM).[5] The structural basis of this affinity enhancement is not evident from the homology model.

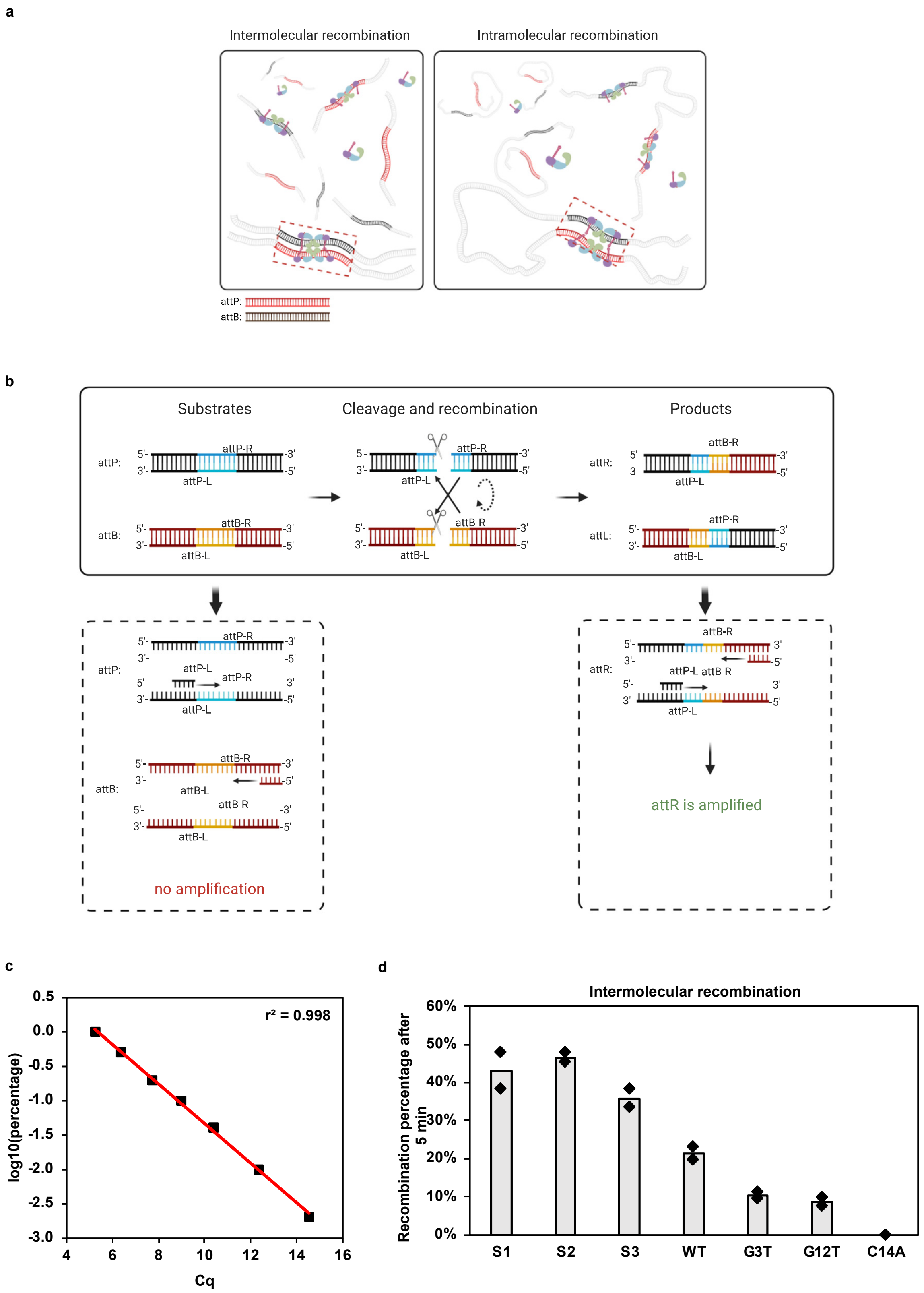

Supplementary Figure 6. Test of selected attP-L sequences in intermolecular DNA recombination *in vitro*. **a** Schematics of intermolecular (left) and intramolecular (right) DNA

recombination, which contrast in their number of substrates but form the same attP-attB-Bxb1 motif (dashed box). **b** qPCR method for quantifying the intermolecular recombination reaction. For the attR product, the black and red primers anneal to two complementary DNA strands, resulting in an exponentially amplified product (right); for the attB and attP substrates (and the attL product), this amplification does not occur (left). **c** Standard curve for qPCR quantification of recombinant attR product yield. **d** Intramolecular recombination test of individual attP-L variants *in vitro*. Bxb1 concentration is 10 nM and both attB and attP substrate concentrations are 10 nM. The reactions were incubated at 30 °C for 5 min and then terminated by heat denaturation at 80 °C for 20 min. The attR reaction product concentration was measured by qPCR. S1: 5A/8T/12C/14T/17A, S2: 5T/12A/17A, S3: 3T/5A/12C/14T/17A. Two biological replicates were performed. Supplementary Figs. 6a and 6b were created with BioRender.com. Source data are provided as a Source Data file.

Supplementary Methods

Supplementary Method 1: *In vivo* selection using *E. coli*

The low copy number vector p15A-P_{cons}-kanR/cmR was used for KanR/CmR reporter plasmid construction and the high copy number pUC19-P_{BAD}-bxb1 vector was used for Bxb1 donor plasmid construction by restriction enzyme digestion and ligation. The primers and plasmids used are listed in Supplementary Tables 1 and 2, respectively.

A two-step transformation method was used to create the selection system. First, the Bxb1 donor plasmid was transformed into TOP10 pro *E. coli* cells. Then, homemade competent cells with the Bxb1 donor plasmid were transformed by the second plasmid p15A-P_{cons}-kanR/cmR, which encoded the attP-L library. After the second transformation step, the cells recovered in 950 µL pre-warmed SOB medium and were then incubated at 37 °C and 200 rpm for 1 h. In order to induce Bxb1 protein expression, 0.1% arabinose was added to the culture. After 45 min or 75 min, the cells were washed twice with SOB medium, and then serially diluted before plating on LB-agar plates with antibiotics (50 µg/ml carbenicillin and 15 ug/ml chloramphenicol). The selection plates were incubated at 37 ºC for 14-16 h and colonies that grew on the selection plates were picked for colony PCR using Phusion High-Fidelity polymerase. The amplicons from individual colonies were Sanger sequenced.

Supplementary Method 2: High-throughput reaction rate characterization *in vitro* using NGS

Species in the *in vitro* DNA library flipping reaction

Species	*Description*	*Initial concentration*
S	total DNA concentration of the attP library	10 nM
S_n	DNA concentration of unflipped attP variant n, n=1~1000000	0.01 pM
P_n	DNA concentration of flipped attP variant n, n=1~1000000	0 pM
E	Bxb1 protein concentration	2-300 nM
ES_n	Complex of Bxb1 and unflipped attP variant n	0 pM

Parameters in the DNA flipping reaction

Species	*Description*
$k_{cat}(n)$	catalyst rate constant for attP variant n
$k_{on}(n)$	binding rate constant for attP variant n
$k_{off}(n)$	dissociation rate constant for attP variant n
$K_d(n) = k_{off}(n)/k_{on}(n)$	equilibrium dissociation constant for the Bxb1-attP(n) complex
$k_{flip}(n)$	DNA flipping rate constant for attP variant n

For attP variant n:

$$S_n + E \underset{k_{off}(n)}{\overset{k_{on}(n)}{\rightleftarrows}} ES_n \xrightarrow{k_{cat}(n)} E + P_n$$

(1) $\frac{dS_n}{dt} = -k_{\text{on}}(n) \cdot S_n \cdot E + k_{\text{off}}(n) \cdot ES_n$

(2) $\frac{dP_n}{dt} = k_{\text{cat}}(n) \cdot ES_n$

(3) $\frac{dES_n}{dt} = k_{\text{on}}(n) \cdot S_n \cdot E - k_{\text{off}}(n) \cdot ES_n - k_{\text{cat}}(n) \cdot ES_n$

(4) $S_n(0) = S_n + ES_n + P_n$

(5) $E(0) = E + \sum ES_n$

In our *in vitro* experiments for quantification of flipping rate, the Bxb1 concentration is 3 nM, the total DNA library concentration is 10 nM, and the wild-type attP-Bxb1 dissociation constant is 60 nM. As shown below, these values allow for two reasonable assumptions: a) quasi-steady state for ES_n and b) $E \approx E(0)$. With these assumptions, the reaction rate can be derived from equation (6) and expressed using equations (7) and (8).

(6) $\frac{dES_n}{dt} = k_{\text{on}}(n) \cdot S_n \cdot E(0) - k_{\text{off}} \cdot ES_n - k_{\text{cat}}(n) \cdot ES_n = 0$

The rate of DNA flipping (dP_n/dt) can be written in terms of a DNA flipping rate constant $k_{flip}(n)$:

(7) $\frac{dP_n}{dt} = k_{\text{flip}}(n) \cdot E(0) \cdot S_n$

where $k_{flip}(n)$ is a function of $k_{on}(n)$, $k_{off}(n)$, and $k_{cat}(n)$:

(8) $k_{\text{flip}}(n) = \frac{k_{\text{cat}}(n) \cdot k_{\text{on}}(n)}{k_{\text{off}}(n) + k_{\text{cat}}(n)}$

For the first assumption, the criteria for the validity of the steady-state approximation is that the time scale for the complex ES to reach steady state, $\tau(ES_n)$, should be much shorter than that of S or P, $\tau(S_n)$. This means that, as species concentrations change over the reaction, at every time point, the ES complex can reach equilibrium rapidly. This criterion requires that equation (9) be satisfied[6]:

(9) $\frac{E(0)}{K_{\text{m}} + S} \ll 1$

This equation holds true for our experimental conditions, since the Bxb1 concentration (3 nM) is much less than the wild-type Bxb1-DNA K_d (60 nM)[5], which is less than K_m since $K_m = K_d + k_{cat}/k_{on}$. Even for attP-L variants in the DNA library with a several-fold increased affinity for Bxb1, the enzyme concentration would still be much lower than this K_d.

Based on the validity of the first assumption, ES_n are at steady state:

(10) $\frac{dES_n}{dt} = k_{\text{on}}(n) \cdot S_n \cdot (E(0) - \sum ES_n) - k_{\text{off}}(n) \cdot ES_n - k_{\text{cat}}(n) \cdot ES_n = 0$

Then, equation (11) can be derived from equation (10):

(11) $\frac{\sum ES_n}{(E(0) - \sum ES_n)} = \sum \left\{ \frac{S_n}{\frac{k_{\text{off}}(n) + k_{\text{cat}}(n)}{k_{\text{on}}(n)}} \right\} < \frac{\sum S_n}{\left[\frac{k_{\text{off}}(n) + k_{\text{cat}}(n)}{k_{\text{on}}(n)} \right]_{\text{min}}}$

In our experiments, $\sum S_n$ = 10 nM, which is considerably smaller than the WT attP-Bxb1 K_d = 60 nM, so the second assumption that $E \approx E(0)$ is also reasonable.

Using this method, the frequency from NGS sequencing was converted to the reaction rate constant k_{flip}, which served as the experimental value for comparison to modeling. In the linear equation (2) in the main text, the objective was to find a set of W_{ij} values that minimized the sum of squares error between the calculated k_{flip} values from equation (2) in the main text and the experimental k_{flip} values determined from NGS frequencies as described here. The regression was implemented in MATLAB with the least squares method and the code is available on GitHub (https://github.com/sarkarlab/Bxb1-attP).

The predicted flipped percentages of individual sequences were calculated using the model-predicted k_{flip} and equation (7).

Supplementary Method 3: Mathematical simulation of GFP and mCherry co-expression in *E. coli*

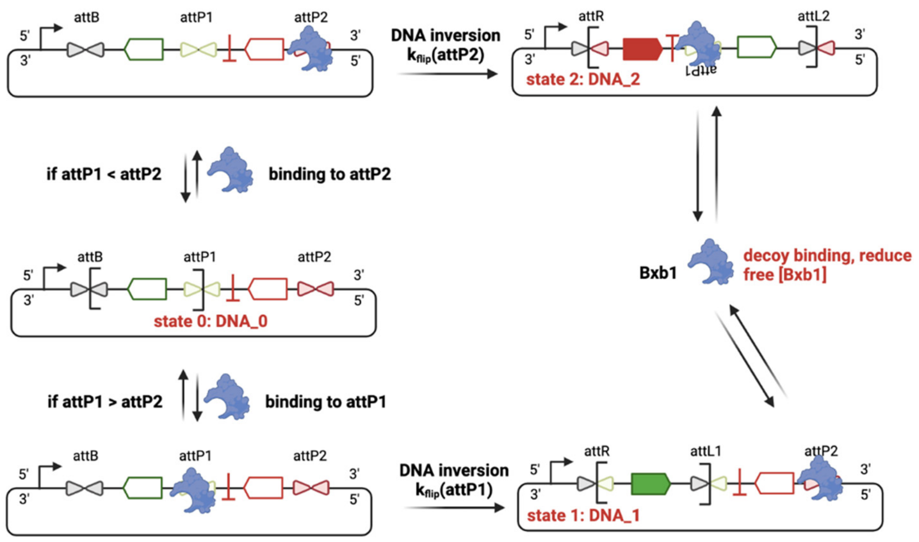

Created with BioRender.com

Species in *E. coli*

Species	*Description*	*Initial concentration*
x	*E. coli* density	1.2×10^{9} cells/ml
DNA_Bxb1	DNA concentration of plasmid DNA coding Bxb1	0.25 nM
DNA_0	unflipped reporter plasmid DNA concentration	25 nM
DNA_1	attP1 flipped reporter plasmid DNA concentration	0 nM
DNA_2	attP2 flipped reporter plasmid DNA concentration	0 nM
mRNA_Bxb1	Bxb1 mRNA concentration	0 nM
mRNA_GFP	GFP mRNA concentration	0 nM
mRNA_mCherry	mCherry mRNA concentration	0 nM
Bxb1	Bxb1 protein concentration	0 nM
GFP	GFP protein concentration	0 nM
mCherry	mCherry protein concentration	0 nM

Parameters in the mathematical model

Parameters	*Description*	*Values*	*References*
k_{flip}(attP1)	DNA flipping rate constant of attP1	0.001-0.01 $nM^{-}min^{-1}$	This study
k_{flip}(attP2)	DNA flipping rate constant of attP2	0.001-0.01 $nM^{-}min^{-1}$	This study
$K_{d.DNA}$(attP1)	Dissociation constant of Bxb1 and attP1	10-1000 nM	[5]
$K_{d.DNA}$(attP2)	Dissociation constant of Bxb1 and attP2	10-1000 nM	[5]
k_m(Bxb1)	Bxb1 mRNA transcription rate constant	1.1 min^{-1}	[7]
k_m(GFP)	GFP mRNA transcription rate constant	2.2 min^{-1}	[7]
k_m(mCherry)	mCherry mRNA transcription rate constant	2.2 min^{-1}	[7]
k_p(Bxb1)	Bxb1 protein translation rate constant	0.32 min^{-1}	[8]
k_p(GFP)	GFP protein translation rate constant	0.66 min^{-1}	[8]
k_p(mCherry)	mCherry protein translation rate constant	0.68 min^{-1}	[8]
k_{deg}(mRNA)	mRNA degradation rate constant	0.14 min^{-1}	[7]
k_{deg}(Bxb1)	Bxb1 protein degradation rate constant	0.012 min^{-1}	[9]
k_{deg}(GFP)	GFP protein degradation rate constant	0.002 min^{-1}	[9]
k_{deg}(mCherry)	mCherry protein degradation rate constant	0.002 min^{-1}	[9]
μ	*E. coli* growth rate	3.6×10^{-3} min^{-1}	This study
K	maximum cell density	2.3×10^{9} cells/ml	This study

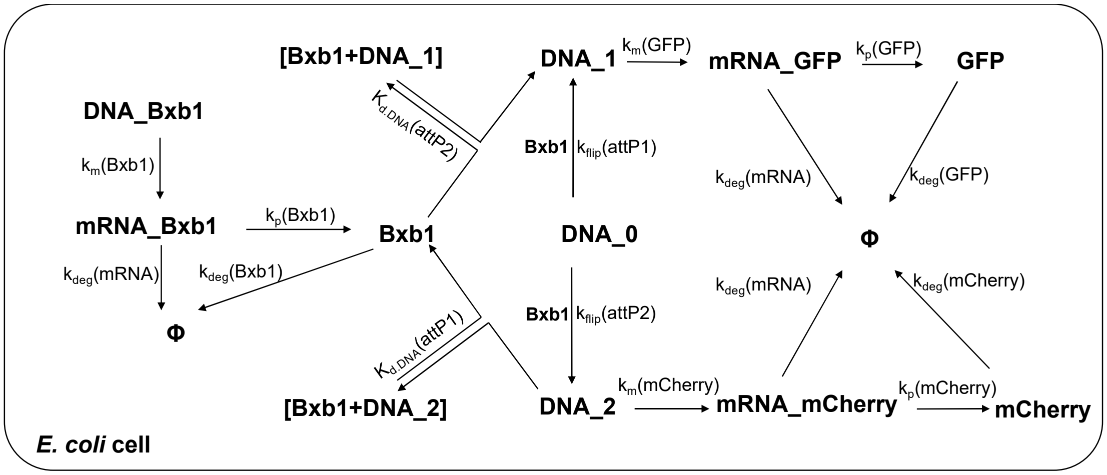

Differential equations describing GFP and mCherry co-expression:

(1) $$\frac{dx}{dt} = \mu \cdot (1 - \frac{x}{K}) \cdot x$$

(2) $$\frac{d(DNA\_Bxb1)}{dt} = -DNA\_Bxb1 \cdot (\mu \cdot (1 - \frac{x}{K}))$$

(3) $$\frac{d(DNA\_1)}{dt} = kflip(attP1) \cdot \frac{Bxb1}{(1 + \frac{DNA\_1}{K_{d\_DNA}(attP2)} + \frac{DNA\_2}{K_{d\_DNA}(attP1)})} \cdot DNA\_0 - DNA\_1 \cdot (\mu \cdot (1 - \frac{x}{K}))$$

(4) $$\frac{d(DNA\_2)}{dt} = kflip(attP2) \cdot \frac{Bxb1}{(1 + \frac{DNA\_1}{K_{d\_DNA}(attP2)} + \frac{DNA\_2}{K_{d\_DNA}(attP1)})} \cdot DNA\_0 - DNA\_2 \cdot (\mu \cdot (1 - \frac{x}{K}))$$

(5) $$\frac{d(DNA\_0)}{dt} = -\frac{d(DNA\_1)}{dt} - \frac{d(DNA\_2)}{dt} - DNA\_0 \cdot (\mu \cdot (1 - \frac{x}{K}))$$

(6) $$\frac{d(mRNA\_Bxb1)}{dt} = k_m(Bxb1) \cdot DNA\_Bxb1 - k_{deg}(mRNA) \cdot mRNA\_Bxb1 - mRNA\_Bxb1 \cdot (\mu \cdot (1 - \frac{x}{K}))$$

(7) $$\frac{d(mRNA\_GFP)}{dt} = k_m(GFP) \cdot DNA\_1 - k_{deg}(mRNA) \cdot mRNA\_GFP - mRNA\_GFP \cdot (\mu \cdot (1 - \frac{x}{K}))$$

(8) $$\frac{d(mRNA\_mCherry)}{dt} = k_m(mCherry) \cdot DNA\_2 - k_{deg}(mRNA) \cdot mRNA\_mCherry - mRNA\_mCherry \cdot (\mu \cdot (1 - \frac{x}{K}))$$

(9) $$\frac{d(Bxb1)}{dt} = k_p(Bxb1) \cdot mRNA\_Bxb1 - k_{deg}(Bxb1) \cdot Bxb1 - Bxb1 \cdot (\mu \cdot (1 - \frac{x}{K}))$$

(10) $$\frac{d(GFP)}{dt} = k_p(GFP) \cdot mRNA\_GFP - k_{deg}(GFP) \cdot GFP - GFP \cdot (\mu \cdot (1 - \frac{x}{K}))$$

(11) $$\frac{d(mCherry)}{dt} = k_p(mCherry) \cdot mRNA\_mCherry - k_{deg}(mCherry) \cdot mCherry - mCherry \cdot (\mu \cdot (1 - \frac{x}{K}))$$

This system of ordinary differential equations was solved in Matlab using ode23s.

Supplementary References

1. Kiefer, F., Arnold, K. & Ku, M. The SWISS-MODEL Repository and associated resources. *Nucleic Acids Res.* **37**, 387–392 (2009).
2. Tuszynska, I., Magnus, M., Jonak, K., Dawson, W. & Bujnicki, M. NPDock : a web server for protein – nucleic acid docking. *Nucleic Acids Res.* **43**, 425–430 (2015).
3. Rutherford, K., Yuan, P., Perry, K., Sharp, R. & Van Duyne, G. D. Attachment site recognition and regulation of directionality by the serine integrases. *Nucleic Acids Res.* **41**, 8341–8356 (2013).
4. Bessen, J. L. *et al.* High-resolution specificity profiling and off-target prediction for site-specific DNA recombinases. *Nat. Commun.* **10**, 1937 (2019).
5. Singh, S., Ghosh, P. & Hatfull, G. F. Attachment Site Selection and Identity in Bxb1 Serine Integrase-Mediated Site-Specific Recombination. *PLoS Genet.* **9**, e1003490 (2013).
6. Segel, L. A. On the validity of the steady state assumption of enzyme kinetics. *Bull. Math. Biol.* **50**, 579–593 (1988).
7. Chen, H., Shiroguchi, K., Ge, H. & Xie, X. S. Genome-wide study of mRNA degradation and transcript elongation in *Escherichia coli*. *Mol. Syst. Biol.* **11**, 781 (2015).
8. Guet, C., Bruneaux, L., Min, T. L., Siegal-gaskins, D. & Figueroa, I. Minimally invasive determination of mRNA concentration in single living bacteria. *Nucleic Acids Res.* **36**, e73 (2008).
9. Maurizi, M. R. Proteases and protein degradation in *Escherichia coli*. *Experientia* **48**, 178–201 (1992).
