# Supplementary material for: Model-guided engineering of DNA sequences with predictable site-specific recombination rates
Source: Nat Commun. 2022 Jul 20;13:4152. doi: 10.1038/s41467-022-31538-3 (PMC9300676; doi:10.1038/s41467-022-31538-3)
Supplement: Supplementary file 2 — Reporting Summary [file 41467_2022_31538_MOESM2_ESM.pdf]

nature portfolio

Corresponding author(s): Casim A. Sarkar

Last updated by author(s): Jun 20, 2022

Reporting Summary

Nature Portfolio wishes to improve the reproducibility of the work that we publish. This form provides structure for consistency and transparency in reporting. For further information on Nature Portfolio policies, see our Editorial Policies and the Editorial Policy Checklist.

Statistics

For all statistical analyses, confirm that the following items are present in the figure legend, table legend, main text, or Methods section.

n/a	Confirmed	
☐	☒	The exact sample size (*n*) for each experimental group/condition, given as a discrete number and unit of measurement
☐	☒	A statement on whether measurements were taken from distinct samples or whether the same sample was measured repeatedly
☒	☐	The statistical test(s) used AND whether they are one- or two-sided<br>*Only common tests should be described solely by name; describe more complex techniques in the Methods section.*
☒	☐	A description of all covariates tested
☒	☐	A description of any assumptions or corrections, such as tests of normality and adjustment for multiple comparisons
☐	☒	A full description of the statistical parameters including central tendency (e.g. means) or other basic estimates (e.g. regression coefficient) AND variation (e.g. standard deviation) or associated estimates of uncertainty (e.g. confidence intervals)
☒	☐	For null hypothesis testing, the test statistic (e.g. *F*, *t*, *r*) with confidence intervals, effect sizes, degrees of freedom and *P* value noted<br>*Give P values as exact values whenever suitable.*
☒	☐	For Bayesian analysis, information on the choice of priors and Markov chain Monte Carlo settings
☒	☐	For hierarchical and complex designs, identification of the appropriate level for tests and full reporting of outcomes
☒	☐	Estimates of effect sizes (e.g. Cohen's *d*, Pearson's *r*), indicating how they were calculated

Our web collection on statistics for biologists contains articles on many of the points above.

Software and code

Policy information about availability of computer code

Data collection	No software was used for data collection.
Data analysis	Matlab (version: R2020b) was used for data analysis and the code is available on GitHub (https://github.com/sarkarlab/Bxb1-attP). SWISS-MODEL (https://swissmodel.expasy.org/) and NPDock (http://genesilico.pl/NPDock) were used to simulate the Bxb1-DNA complex.

For manuscripts utilizing custom algorithms or software that are central to the research but not yet described in published literature, software must be made available to editors and reviewers. We strongly encourage code deposition in a community repository (e.g. GitHub). See the Nature Portfolio guidelines for submitting code & software for further information.

Data

Policy information about availability of data

All manuscripts must include a data availability statement. This statement should provide the following information, where applicable:

- Accession codes, unique identifiers, or web links for publicly available datasets
- A description of any restrictions on data availability
- For clinical datasets or third party data, please ensure that the statement adheres to our policy

High-throughput sequencing data have been deposited in the NCBI Sequence Read Archive database under accession number PRJNA752141 (https://www.ncbi.nlm.nih.gov/bioproject/PRJNA752141/). Source data are provided with this paper.

The Bxb1 protein structure (PDB: 4KIS) was used as a template for simulating the Bxb1-DNA complex.

Field-specific reporting

Please select the one below that is the best fit for your research. If you are not sure, read the appropriate sections before making your selection.

- [x] Life sciences
- [ ] Behavioural & social sciences
- [ ] Ecological, evolutionary & environmental sciences

For a reference copy of the document with all sections, see nature.com/documents/nr-reporting-summary-flat.pdf

Life sciences study design

All studies must disclose on these points even when the disclosure is negative.

Sample size	Sequencing depth was determined by the sequencing capacity of the Illumina MiniSeq instrument, with a minimum average of 400,000 reads per NGS experiment, which provided sufficient data quality.
Data exclusions	When analyzing NGS data, attP variants with mismatches in non-randomized positions were excluded, as we determined that these sequence variants were typically due to sequencing errors.
Replication	At least three technical replicates were performed for in vitro assays, and at least three biological replicates were performed for measurements in E. coli. All attempts at replication were successful.
Randomization	Randomization was not employed since identical experimental conditions were used for each in vitro or cell-based assay (outside of the variable of interest).
Blinding	Blinding was not employed since identical experimental conditions were used for each in vitro or cell-based assay (outside of the variable of interest).

Reporting for specific materials, systems and methods

We require information from authors about some types of materials, experimental systems and methods used in many studies. Here, indicate whether each material, system or method listed is relevant to your study. If you are not sure if a list item applies to your research, read the appropriate section before selecting a response.

Materials & experimental systems

n/a	Involved in the study	
x		Antibodies
x		Eukaryotic cell lines
x		Palaeontology and archaeology
x		Animals and other organisms
x		Human research participants
x		Clinical data
x		Dual use research of concern

Methods

n/a	Involved in the study	
x		ChIP-seq
x		Flow cytometry
x		MRI-based neuroimaging
